# Intelligent Gearbox Diagnosis Methods Based on SVM, Wavelet Lifting and RBR

**DOI:** 10.3390/s100504602

**Published:** 2010-05-04

**Authors:** Lixin Gao, Zhiqiang Ren, Wenliang Tang, Huaqing Wang, Peng Chen

**Affiliations:** 1 Key Laboratory of Advanced Manufacturing Technology, Beijing University of Technology, Chao Yang District, Beijing, 100124, China; E-Mails: lead0003@163.com (L.X.G.); renzhiqiang1221@163.com (Z.Q.R.); twllxy@emails.bjut.edu.cn (W.L.T.); 2 School of Mechanical & Electrical Engineering, Beijing University of Chemical Technology, Chao Yang District, Beijing, 100029, China; 3 Graduate School of Bioresources, Mie University, 1577 Kurimamachiya-cho, Tsu, Mie, 514-8507, Japan; E-Mail: chen@bio.mie-u.ac.jp

**Keywords:** gearbox, support vector machines (SVM), wavelet lifting, rule-based reasoning (RBR), intelligent diagnosis

## Abstract

Given the problems in intelligent gearbox diagnosis methods, it is difficult to obtain the desired information and a large enough sample size to study; therefore, we propose the application of various methods for gearbox fault diagnosis, including wavelet lifting, a support vector machine (SVM) and rule-based reasoning (RBR). In a complex field environment, it is less likely for machines to have the same fault; moreover, the fault features can also vary. Therefore, a SVM could be used for the initial diagnosis. First, gearbox vibration signals were processed with wavelet packet decomposition, and the signal energy coefficients of each frequency band were extracted and used as input feature vectors in SVM for normal and faulty pattern recognition. Second, precision analysis using wavelet lifting could successfully filter out the noisy signals while maintaining the impulse characteristics of the fault; thus effectively extracting the fault frequency of the machine. Lastly, the knowledge base was built based on the field rules summarized by experts to identify the detailed fault type. Results have shown that SVM is a powerful tool to accomplish gearbox fault pattern recognition when the sample size is small, whereas the wavelet lifting scheme can effectively extract fault features, and rule-based reasoning can be used to identify the detailed fault type. Therefore, a method that combines SVM, wavelet lifting and rule-based reasoning ensures effective gearbox fault diagnosis.

## 1. Introduction

With the continuous development of modern industrial large-scale manufacturing and progress in the sciences and technology, machinery, as the major production tool, tends to be large, complex, speedy, continuous and automatic to maximally improve production efficiency and product quality. Machine production efficiency is increasing, and their mechanical structures are becoming more complicated. Once a machine breaks down, the whole production process must stop, which can lead to enormous economic losses and serious personnel injuries. Therefore, reliable and safe equipment operation is required. It has been proved that constantly monitoring equipment conditions and effectively implementing fault diagnosis techniques are the major preventive measures that guarantee safe equipment operation by detecting faults at an early stage to avoid major and fatal accidents.

An intelligent machine fault diagnosis system has been developed rapidly in the past decades by successfully applying new theories. Meanwhile, the large scale and complexity of modern machines, together with the urgent needs of real-time and automatic machine fault diagnosis, have driven the transformation of fault diagnosis technology from artificial diagnosis to intelligent diagnosis.

Among all kinds of intelligent diagnosis methods, pattern recognition based on an Artificial Neural Network (ANN) has been widely used because of its power in self- organizing, unsupervised-learning, and nonlinear pattern classification [1]. However, in practice, it is difficult to obtain the large quantity of typical fault samples that is required by an ANN. Because machinery malfunctions, especially large-scale machinery and equipment malfunctions, can lead to huge economic losses, few fault samples are available. Thus, these fault diagnosis methods, although excellent in theory, do not perform well in practice [2]. The newly developed support vector machine (SVM) opens a new path to resolve problems in fault diagnosis. The SVM was originally proposed by Vapnik in 1979. It is a new tool that supports machine learning with an optimal approach and has shown unique advantages and a promising future in resolving small sample size issues in pattern recognition. SVMs specifically target the issue of limited samples and aim to obtain the optimal solution with the available information, rather than the optimal value with a sample size close to infinity; its algorithm is converted to a quadratic optimization problem and, theoretically, produces a globally optimal solution, which solves the inevitable local extrema problem in neural network methods. The algorithm transforms the problem to a high-dimensional eigenspace through a kernel-based nonlinear transformation, and a linear discriminant function is subsequently constructed in high-dimensional space to achieve the nonlinear discriminant function in the original space. Thus, the dimension problem is solved cleverly and the complexity of the algorithm is independent of the sample dimension [3]. Although the support vector data are significantly lower than the number of training samples, there are still some problems. For example, the support vector data grow linearly with the number of training samples, which may lead to excessive fitting and is time-consuming in calculation; probability prediction can not be obtained with a SVM; users of SVM must give an error parameter, which significantly influences the results. Unfortunately, the value of the given parameter is highly subjective, and all its possible values have to be guessed in order to find the best result. Moreover, the kernel function of SVM must fulfill Mercer’s condition [4].

It is well known that the bottleneck of fault diagnosis is a lack of fault samples, which provides SVM a bright application future in machine fault diagnosis. Jack has used SVM to detect the rolling bearing condition [5]; he also optimized the SVM parameter with a genetic algorithm and achieved a good generalization [6]. Thukaram *et al*. compared the differences between neural networks and SVMs in recognizing faults, and demonstrated the advantages of SVM in situations with small sample size. Nonetheless, most studies are still limited in laboratory tests; there are not many applications of SVM in intelligent fault diagnosis systems in practice. More research and field tests are required for application of SVM in practice. We investigate further in this field.

The wavelet transform is a breakthrough in signal processing technology in the past two decades [7]. Currently, the wavelet lifting analysis algorithm has been successfully applied in many fields, even though it was only recently proposed. Calderbank, Daubechies and Sweldens *et al*. have applied wavelet lifting analysis to the image compressing field and have achieved better compression compared to first generation wavelet analysis [8,9]. Howlet and Nguyen have applied the wavelet lifting transform to audio signal analysis to reduce the signal Shannon entropy by approximately 6% [10]. MIT investigators Sudarshan *et al*. combined the wavelet lifting transform with finite element analysis and proposed a novel multiresolution finite element method. In fault diagnosis, application of the wavelet lifting transform has just begun. Based on Claypoole’s self-adaptive wavelet transformation, Samuel and Pines at the University of Maryland developed a new method using the wavelet lifting combined with matching pursuit gear fault features, which has led to satisfactory results in helicopter transmission fault diagnostics [11]. Zhengjia He and Chendong Duan *et al*. in Xi’an Jiaotong University have also done considerable research in this field. They have deduced several construction methods of wavelet lifting and obtained excellent analysis results in signal processing, time-frequency analysis and feature extraction when combining wavelet lifting with other methods [8,12].

Rule-based reasoning (RBR) is a traditional intelligent diagnosis method. Experience and knowledge will be represented in the form of rules which will be saved in knowledge base, and the reasoning mechanisms will be used to get the diagnosis conclusions with the rules. Considering the engine wear process, Peilin Zhang, Bing Li and Shubao Liang *et al*. have established a fuzzy rule based on typical wear faults for certain engines. They have introduced a symmetric fuzzy cross-entropy method for fault reasoning and established a model of engine fault diagnosis based on a combined method of symmetric fuzzy cross-entropy and rule-based reasoning [13]. In order to perform a flexible, rapid and precise case adaptation in a case-based reasoning design system, Xin Song, Wei Guo and Zhiyong Wang have proposed a case adaptation mechanism that is based on regression analysis and rule-based reasoning [14].

**Figure 1.**
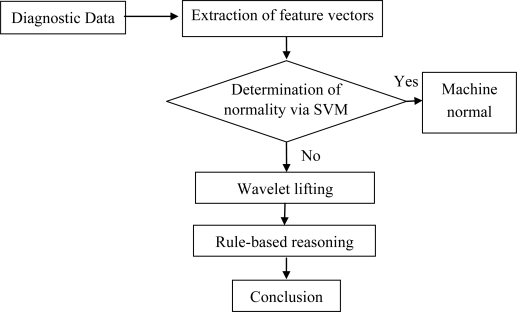
The principle of intelligent fault diagnosis based on SVM, wavelet lifting and RBR.

This study presents a method that combines wavelet lifting, an SVM and rule-based reasoning to diagnose gearbox faults. Gearbox vibration signals are initially processed by wavelet packet decomposition. Then, the energy coefficients of each frequency band are calculated and used as input vectors to the SVM to recognize normal and faulty gearbox patterns. Precise analysis from the wavelet lifting scheme was then utilized to obtain the machine fault feature frequency. Finally, based on the fault feature frequency, the existing diagnostic knowledge and rules were used for logical reasoning to establish a knowledge base to identify fault types. The diagnosis scheme based on an SVM, wavelet lifting and rule-based reasoning methods is shown in Figure 1.

## 2. Application of SVM in Machine Fault Diagnosis

### 2.1. Principle of SVM

A support vector machine is based on minimizing structural risks. Its algorithm was initially designed for two-class classification. In the field of machine faults, an SVM can simply determine whether there is a fault.

The SVM method is developed by determining the optimal separating hyperplane in for linear separability. The optimal separating hyperplane is not only able to classify all training samples, but also maximizes the distance between the separating hyperplane and points in training samples that are closest to the separating plane.

The fault training sample set is (*x_i_, y_i_*), *i* = 1,…, *n, x* ∈ *R^d^*, *y* ∈{ +1, −1}, where n is the number of training samples, and d is the dimension of fault feature vectors. Assuming the following equation is satisfied:

(1) $$y_{i}\left[\left({wx}_{i}\right)+b\right]\geq 1,i=1,...,n$$

the minimum value of *φ*(*w*) is:

(2) $$\varphi (w)=\frac{1}{2}{\left\|w\right\|}^{2}=\frac{1}{2}(\mathrm{ww})\;$$

The solution of this optimal problem is the saddle point of the Lagrange function, and the optimal discriminant function is obtained as:

(3) $$f(x)=\text{sgn}\left[\left(w^{*}x\right)+b^{*}\right]=\text{sgn}\sum_{i=1}^{n}\alpha_{i}^{*}y_{i}\left[\left(x_{i}x\right)+b^{*}\right]$$

Nonlinear problems can be converted to high-dimensional linear problems with a nonlinear transformation. In high-dimensional space, only the inner-product computation is needed, which can be obtained by using functions in the original low dimensions. According to the relative principles of functional analysis, if one kernel function K(*x_i_*,*x*) fulfills Mercer’s conditions, it corresponds to the inner product of one dimension. Such functions are called kernel functions, and the optimal discriminant function in this situation is changed to:

(4) $$f(x)=\text{sgn}\left(\sum_{i=1}^{n}\alpha_{i}^{*}y_{i}K\left(x_{i},x\right)+b^{*}\right)$$

The kernel functions commonly used are the RBF kernel, MLP kernel and Multinomial kernel. However, the RBF described as $K\left(x_{i},x\right)=\text{exp}\left\{-\frac{{\left|x_{i}-x\right|}^{2}}{\sigma^{2}}\right\}$ is used most widely. It will be described in detail in reference [16]. Because the RBF kernel performs better in recognition than the MLP kernel or Multinomial kernel, and the SVM algorithm has higher recognition accuracy and is more suitable than a BP neural network to deal with a small sample data set [15,16], this study employs the RBF kernel in the LS-SVM toolbox to diagnose the fault.

### 2.2. Feature vector analysis of wavelet packets energy

Using multiresolution analysis and the wavelet packet technique, signals can be decomposed into different frequency bands. Analyzing signals in these frequency bands is called frequency bandwidth analysis. Usually, based on the frequency range where signals of interest are located, users can decompose signals to a certain scale and obtain information from the corresponding frequency bands. Additionally, signals in different frequency bands can be further subject to statistical analyses to obtain feature vectors that represent signal characteristics. Analyzing the signal energy in different frequency bands is called frequency band energy analysis. It is characterized by wide-frequency-range responses when processing nonstationary, transient signals with higher frequency resolution at low frequency and higher time resolution at high frequency. Compared to the FFT, it contains a great deal of non-stationary and nonlinear diagnostic information.

The theoretical basis for wavelet frequency bandwidth analysis is Parseval’s theorem. The time domain energy of *f(x)* is ${\left\|f\right\|}^{2}=\int_{R}{\left|f(x)\right|}^{2}\mathrm{dx}$; the wavelet transform of *f(x)* is $d(j,k)=W(2^{j},2^{j}k)=2^{\frac{-j}{2}}\int_{R}\bar{\psi }(2^{-j}x-k)f(x)dx$; and these two are linked by Parseval’s equation:

(5) $$\int_{R}{\left|f(x)\right|}^{2}\mathrm{dx}=\sum_{j}|d_{\mathrm{jk}}{|}^{2}$$

Thus, vibration signals are decomposed into independent frequency bands of different levels by using a conjugate quadrature filter. Not only are these decomposed signals in quadrature to each other in agreement with the law of conservation of energy, but they also contain a large quantity of non-stationary and nonlinear diagnostic information compared to an FFT. Therefore, the signal energy in every frequency band can be used as a feature vector to represent the operation condition of the machine and is useful for machine fault diagnosis.

The procedure for feature vector extraction using wavelets is the following:

Step 1: process vibration signals for wavelet packet decomposition;
Step 2: reconstruct each wavelet packet coefficient, and extract signals in different frequency ranges;
Step 3: acquire *E_j_*, the signal energy at different frequency bands and the total energy *E*. $E_{j}=\sum_{k=1}^{n}{\left|x_{\mathrm{jk}}\right|}^{2}$ and $E=\sum_{j=1}^{l}E_{j}$, where *k* = 1, 2, 3…n is defined as the discrete points at frequency band *j*; *j* is the number of frequency bands; and *x_jk_* represents the amplitude of the discrete points.
Step 4: use the percentile ratio of the signal energy *E_j_* at each decomposed frequency band and the total energy *E* as elements to construct feature vectors.

## 3. Wavelet Lifting Scheme

**Figure 2.**
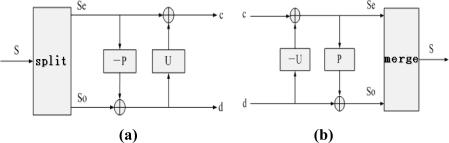
Decomposition and reconstruction process of the wavelet lifting. (a) decomposition of the wavelet lifting; (b) reconstruction of the wavelet lifting.

The wavelet lifting transform includes two stages: decomposition and reconstruction. Decomposition consists of splitting, predicting and updating. As shown in Figure 2(a), given data series *S*= {*s*(*k*), *k* ∈ *Z*}, the decomposition stage of the wavelet lifting transform based on the lifting scheme is shown below:

1. Split: the data series {*s*(*k*), *k* ∈ *Z*} is split into an odd sample series *s_o_*(*k*) and even sample series *S_e_*(*k*)
  (6) $$s_{o}(k)=s(2k+1)\;k\in Z$$
  (7) $$s_{e}(k)=s(2k)\;k\in Z$$
2. Predict: suppose *P*(•) is the predictor; then use *s_e_*(*k*) to predict *s_o_*(*k*), and define the predictive deviation as the detail signal *d*(*k*):
  (8) $$d(k)=s_{o}(k)-P\left[s_{e}(k)\right]\;k\in Z$$
  Then, the detail signal series is *D* = {*d*(*k*), *k* ∈ *Z*}
3. Update: assume *U*(•) is the updater, then *s_e_*(*k*) is updated based on the detail signal *d*(*k*). Its result is defined as the approximation signal *c*(*k*):
  (9) $$c(k)=s_{e}(k)+U[d(k)]\;k\in Z$$
  Then, the approximation signal series is *C* = {*c*(*k*), *k* ∈ *Z*}

Reconstruction of the wavelet lifting is the reverse process of decomposition, and is composed of recovery prediction, recovery updating and merging:

(10) $$s_{e}(k)=c(k)-U[d(k)]\;k\in Z$$

(11) $$s_{o}(k)=d(k)+P[s_{e}(k)]\;k\in Z$$

The reconstruction signal s is obtained by merging the odd and even sample series, as shown in Figure 2 (b).

## 4. Fundamental Ideas of RBR Diagnostic Strategy

### 4.1. Fuzzy reasoning mechanism of typical faults in RBR

Because of the differences in machine working conditions, vibration signals can provide significant qualitative information. However, there is no one-to-one correspondence between fault features and conclusions because of the complexity of the machines. Therefore, in the diagnosis system used in this study, the fuzzy reasoning strategy was used to perfect rule-based diagnosis methods. The knowledge base is represented by the production rule. The fundamental ideas of fuzzy reasoning are as follows:

Suppose G is a set of a fuzzy proposition, fuzzy characteristics and a fuzzy relation. For simplicity, the fuzzy proposition, fuzzy characteristics and fuzzy relation are together called the fuzzy assertion. Then, a piece of factual information can be presented by a binary group (*P*, *β*).

*P* is the fuzzy assertion, *P* ∈ *G; β* is the reliability of *P*, β ∈ [0, 1].

One fault symptom may correspond to multiple causes, while one fault cause may also correspond to multiple fault symptoms. Therefore, the relationship between cause and symptom is complicated.

For proper diagnosis, the membership degree between fault causes and fault symptoms needs to be pre-determined. The value of this membership degree can be obtained based on expert experience or theoretical research. Based on years of experience in our lab in field diagnosis, we summarize the rules and establish the knowledge base.

The fuzzy rules of the knowledge base were used for fault cause reasoning to determine the reason for the faults. Then, according to the typical gearbox fault features, the rules of the knowledge base are constructed as shown in Table 1. In Table 1, *f_r_*, *f_m_* and *x_q_* are rotation frequency, gear meshing frequency and kurtosis, respectively.

For gearbox fault diagnosis, a fuzzy matrix was established:

(12) $$R=\left[\begin{array}{cccccccccccc} 0.8 & 0 & 0 & 0 & 0 & 0 & 0 & 0 & 0 & 0.2 & 0 & 0\\ 0.4 & 0.3 & 0.2 & 0 & 0 & 0 & 0 & 0 & 0 & 0 & 0.1 & 0\\ 0.7 & 0 & 0 & 0 & 0 & 0 & 0 & 0 & 0 & 0.3 & 0 & 0\\ 0.4 & 0.2 & 0.2 & 0 & 0 & 0 & 0 & 0 & 0 & 0 & 0.2 & 0\\ 0.2 & 0.1 & 0.1 & 0 & 0 & 0.2 & 0.1 & 0.1 & 0.1 & 0.1 & 0 & 0\\ 0 & 0 & 0 & 0 & 0 & 0.4 & 0.2 & 0.2 & 0.1 & 0.1 & 0 & 0\\ 0.2 & 0.1 & 0 & 0 & 0 & 0.2 & 0.1 & 0.1 & 0.2 & 0 & 0 & 0.1\\ 0.4 & 0.1 & 0.1 & 0.1 & 0.1 & 0 & 0 & 0 & 0.2 & 0 & 0 & 0 \end{array}\right]$$

In the fuzzy matrix for gearbox fault diagnosis, rows represent sets of fault causes, columns represent sets of faults symptoms, and the values in the matrix represent the membership degree between fault symptoms and causes.

When implementing fault diagnosis with the fuzzy reasoning approach, the fuzzy matrix *R* is established first. Given a fault symptom *A*, if the fault conclusion is *B*, then the fuzzy reasoning formula can be shown as the following:

(13) $$B=R*A=\left[\begin{array}{cccc} r_{11} & r_{12} & ... & r_{1m}\\ r_{21} & r_{22} & ... & r_{2m}\\ ... & ... & ... & ...\\ r_{n1} & r_{n2} & ... & r_{\mathrm{nm}} \end{array}\right]*\left[\begin{array}{l} a_{1}\\ a_{2}\\ ...\\ a_{m} \end{array}\right]$$

The final diagnosis result includes the vectors with relatively large values upon conclusion of the diagnosis. If there are several relatively large values, the existing fault symptom should be considered for the final conclusion.

## 5. Examples of Diagnosis

**Figure 3.**
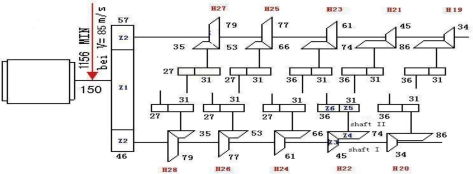
The gearbox transmission chain chart.

In this study, SVM, wavelet lifting and diagnosis rules were used to analyze the vibration acceleration signal, according to a broken cog fault of the Z5 gear (tooth 31) in Shaft II of 22 gear-boxes of a high speed wire rolling mill. The gearbox transmission chain of a high-speed wire rolling mill is shown in Figure 3.

**Figure 4.**
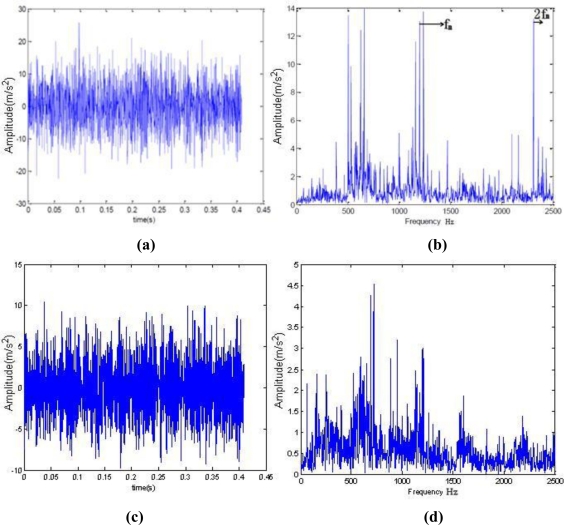
Comparison between waveform and spectrum with fault or fault-free. (a) time-domain waveform when the fault occurred; (b) spectrum when the fault occurred; (c) time domain waveform under normal condition; (d) spectrum under normal condition.

Figure 4(a) shows the broken cog fault signals of one rolling mill. The fault happened in November 2006. The motor was running at 1,169 r/min. The sampling frequency was 5,000 Hz, the sampling number was 2,048, and the Z5/Z6 meshing frequency was 1,197.676 Hz.

As seen in Figure 4(b), the spectrum on the day of the fault shows single frequency and double frequency, and the amplitude of the single frequency is very high, which indicates a severe gear problem at that time. Figure 4(c) shows the time domain waveform under normal condition and Figure 4(d) shows spectrum under normal condition.

### 5.1. SVM estimation

As mentioned in Section 2.2, the “db10” wavelet was used to decompose the signal into three layers and the energy of each of the eight decomposed frequency bands *E_j_* and the total energy *E* were acquired. The feature vector can be established using the ratio between *E_j_* and *E*. The horizontal axis represents energy of each of the eight frequencies after the signals have been decomposed. The vertical axis represents the ratio.

**Figure 5.**
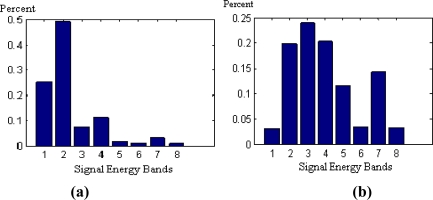
Wavelet energy. (a) normal wavelet energy profile; (b) faulty wavelet energy profile.

As shown in Figure 5, wavelet energy was concentrated in frequency bands 1 and 2 under normal conditions, and tended to move to higher frequency bands when a fault occurred.

**Figure 6.**
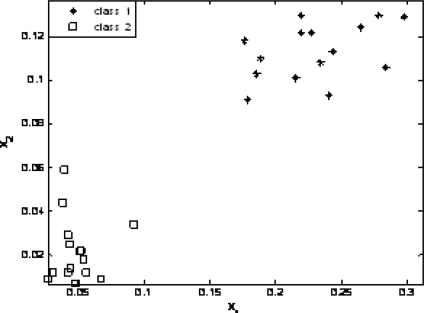
SVM Test Result.

The wavelet energy from the hourly data obtained in early June is set to class 1, which indicates normal conditions; the wavelet energy from data obtained when the rolling mills experienced a fault is set to class 2. Fifteen sets of data were used as SVM input for training. The test data included data from June and September, and each had 15 sets of data. Because gears have different crack patterns, data from September were identified as fault class 2 by the SVM and were significantly different from data in the normal condition. Figure 6 shows the test results.

### 5.2. Wavelet lifting analysis

**Figure 7.**
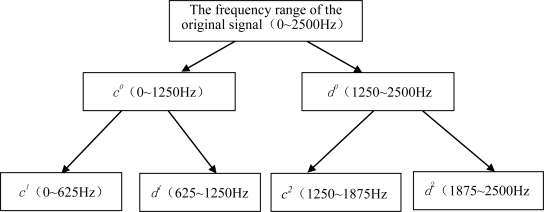
Decomposition of the original signals (the spectrum range 0–2500Hz) through wavelet lifting.

**Figure 8.**
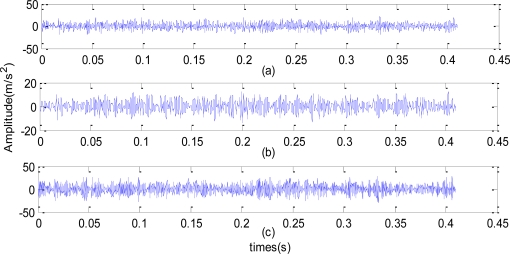
The time domain signals obtained at 1:00 on September 1st, 2006 through wavelet lifting analysis. Approximation signal and detail signal after decomposition through wavelet lifting at level 2. (a) the approximation coefficient *c*^1^; (b) the approximation coefficient *c*^2^; (c) the detail coefficient *d*^2^.

In order to verify the effectiveness of wavelet lifting on data analysis, the field data obtained 74 days before the machine malfunction were analyzed. Through wavelet lifting, the original signals with the spectrum ranging from 0 to 2,500 Hz were decomposed at two levels, as shown in Figure 7. Two different bands can be obtained after carrying out decomposition at level 1, among which the spectrum range of *c*^0^ is 0∼1,250 Hz, and that of *d*^0^ is 1,250∼2,500 Hz. Four different bands can be obtained after carrying out decomposition at level 2, among which the spectrum range of *c*^1^ is 0∼625 Hz, that of *d*^1^ is 625∼1,250 Hz, that of *c*^2^ is 1,250∼1,875 Hz, and that of *d^2^* is 1,875∼2,500 Hz. The approximation coefficient of the wavelet lifting decomposition at level 2 *c^1^*, the approximation coefficient of the wavelet decomposition at level 2 *c*^2^, and the detail coefficient of the wavelet decomposition at level 2 *d*^2^ are shown in Figure 8. The approximation coefficient of wavelet lifting decomposition contains the low frequency information of the signals, and the detail coefficient contains the high frequency information of the signals.

According to the rotational speed of motor, the frequency of all parts in a rolling mill can be calculated, among which *f_m_*, the meshing frequency of high speed axis gear pair Z5/Z6 is 1,140 Hz, and the double frequency is 2,280.4 Hz. Both of the frequencies are included in the reconstructed spectrum *d*^1^ (625∼1,250 Hz) and *d*^2^ (1,875∼2,500 Hz) respectively, after decomposition at level one and level two. Thus the wavelet lifting only at level one and two are decomposed without any other more decompositions in this paper.

**Figure 9.**
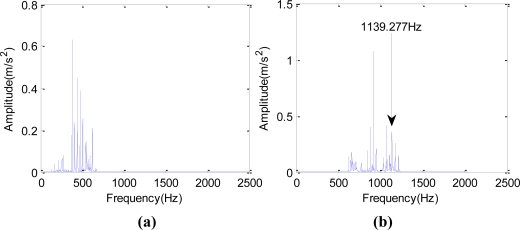

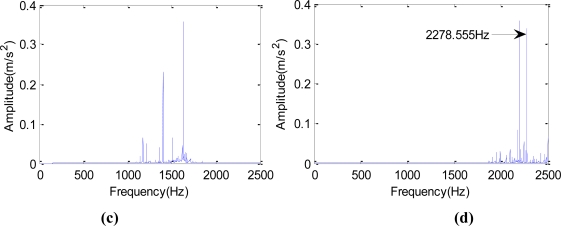
The time domain signals obtained at 1:00 on September 1st, 2006 through wavelet lifting analysis. (a) the spectrum range of *c*^1^ is 0∼625 Hz; (b) the spectrum range of *d*^1^ is 625∼1,250 Hz; (c) the spectrum range of *c*^2^ is 1,250∼1,875 Hz; (d) the spectrum range of *d*^2^ is 1,875∼2,500 Hz.

**Figure 10.**
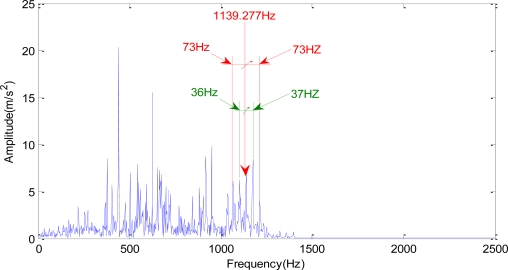
Low frequency spectrum (0∼1,250 Hz) of wavelet lifting level 1 decomposition (with the spectrum of *c*^0^ ranging from 0∼1,250 Hz).

The spectrum obtained from autoregressive spectrum analysis of signals in Figure 8 after wavelet lifting decomposition and reconstruction at level 2 is shown in Figure 9. The single frequency *f_m_* is 1,139.277 Hz, as shown in Figure 9(b), and the double frequency of the Z5/Z6 gear meshing frequency (*f_m_*) can be found in Figure 9(d). The spectrum of signals (the spectrum of *c*^0^ is 0∼1,250 Hz) obtained by reconstruction of the approximation signal after wavelet lifting decomposition at level 1 is shown in Figure 10. There are the gear meshing frequency of the Z5/Z6-1,139.277 Hz, and the side frequencies of 37 Hz and 73 Hz around *f_m_*. The side frequencies of 37 Hz and 73 Hz are close to the single frequency and double frequency, respectively, of 36.751 Hz, which is the shaft-frequency of Shaft II.

### 5.3. Rule-based Reasoning analysis

Through wavelet lifting analysis, the gear meshing frequency (*f_m_*) of Z5/Z6 and shaft-frequency (*f_r_*) of Shaft II are obtained 74 days before the machine malfunction. The ratio of the calculated frequency to the feature frequency is shown in Table 2.

Here, *f_m_* indicates the gear meshing frequency of Z5/Z6. The calculated frequency is 1,140 Hz obtained through rotational speed of field motor, while the feature frequency is 1,139.277 Hz obtained through monitoring spectrum. *f_r_* means shaft-frequency. The calculated frequency is 37.00 Hz obtained through rotational speed of the motor in the field, while the feature frequency is 36.751 Hz obtained through monitoring spectrum.

In Figure 10, there are many 37 Hz side frequencies around *f_m_*, which is 1,139.277 Hz representing the gear meshing frequency. These frequencies are defined as side frequency. In this case, the side frequency (37 Hz) is very close to the shaft-frequency (36.751 Hz) of Shaft II.

The ratio can be obtained through the following calculation. The closer this ratio is to 1, the more consistent is the feature frequency of the monitoring spectrum with the calculating frequency of the fault part, and the more possibility there is of a part with some fault. The process of calculation is shown as follows: 1,139.277/1,140.00 = 0.999, 36.751/37.00 = 0.993.

By analyzing September data in Table 2, it is noted that the single and double frequencies of the Z5/Z6 meshing frequency, as well as the double shaft-frequency of Shaft II, are outstanding. The calculated kurtosis is greater than 6. Compared to fault symptoms with kurtosis greater than 3, fault symptoms with a kurtosis over 3 are very likely. The *f_m_*, *2f_m_*, *f_r_* and peak values are 0.2337, 0.3037, 0.5685 and 0.9855 respectively. The ratio of the fm to the peak value is about 0.237, which is smaller than 0.4. The symptom can be quantified by combining the above values, and the phasor of this fault symptom A = [0.99, 0.99, 0, 0, 0, 0.99, 0.99, 0, 0.95, 0, 0, 0.9].

The calculation of the fault conclusion phasor B is shown below:

$$B=R=\left[\begin{array}{cccccccccccc} 0.8 & 0 & 0 & 0 & 0 & 0 & 0 & 0 & 0 & 0.2 & 0 & 0\\ 0.4 & 0.3 & 0.2 & 0 & 0 & 0 & 0 & 0 & 0 & 0 & 0.1 & 0\\ 0.7 & 0 & 0 & 0 & 0 & 0 & 0 & 0 & 0 & 0.3 & 0 & 0\\ 0.4 & 0.2 & 0.2 & 0 & 0 & 0 & 0 & 0 & 0 & 0 & 0.2 & 0\\ 0.2 & 0.1 & 0.1 & 0 & 0 & 0.2 & 0.1 & 0.1 & 0.1 & 0.1 & 0 & 0\\ 0 & 0 & 0 & 0 & 0 & 0.4 & 0.2 & 0.2 & 0.1 & 0.1 & 0 & 0\\ 0.2 & 0.1 & 0 & 0 & 0 & 0.2 & 0.1 & 0.1 & 0.2 & 0 & 0 & 0.1\\ 0.4 & 0.1 & 0.1 & 0.1 & 0.1 & 0 & 0 & 0 & 0.2 & 0 & 0 & 0 \end{array}\right]*\left[\begin{array}{l} 0.99\\ 0.99\\ 0\\ 0\\ 0\\ 0.99\\ 0.99\\ 0\\ 0.95\\ 0\\ 0\\ 0.9 \end{array}\right]=\left[\begin{array}{l} 0.792\\ 0.693\\ 0.693\\ 0.594\\ 0.689\\ 0.689\\ 0.874\\ 0.685 \end{array}\right]$$

As calculated in the final result, the maximum value that the fault conclusion corresponds to is 0.874; thus, the corresponding fault cause can be confirmed to be a broken gear tooth. A broken cog was found in gearbox Z5 when the machine was disassembled in the field, which is consistent with the diagnosis conclusion.

Together with the fuzzy reasoning approach in the above fault, we have proved that, in fault diagnosis, the application of fuzzy logic can effectively present some fuzzy information and construct a fuzzy matrix; furthermore, the fault type can be effectively diagnosed with fuzzy reasoning.

### 5.4. Analysis of the fault diagnosis ability

In order to describe the ability of intelligent diagnosis method put forward in this paper, we carried out two cases and made comparative analysis with traditional method of Fourier transform.

#### 5.4.1. Case 1: fault diagnosis for tooth collision of helical gear

**Figure 11.**
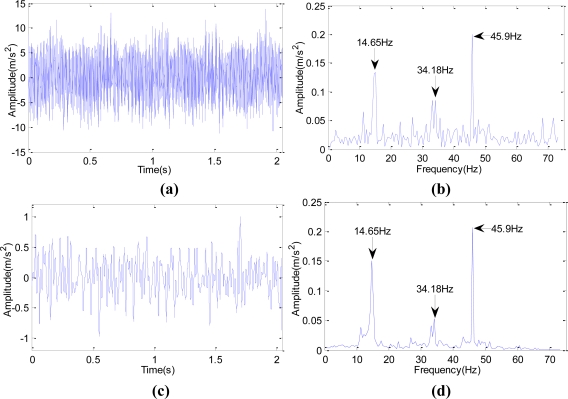
Comparison on the spectrum of Fourier Transform and wavelet lifting analysis of the original signals. (a) time domain waveform of the original signals; (b) spectrum of the original signals after Fourier Transform; (c) time domain waveform of approximation signal reconstruction after decomposition of wavelet lifting at level three; (d) spectrum analysis of wavelet lifting.

**Figure 12.**
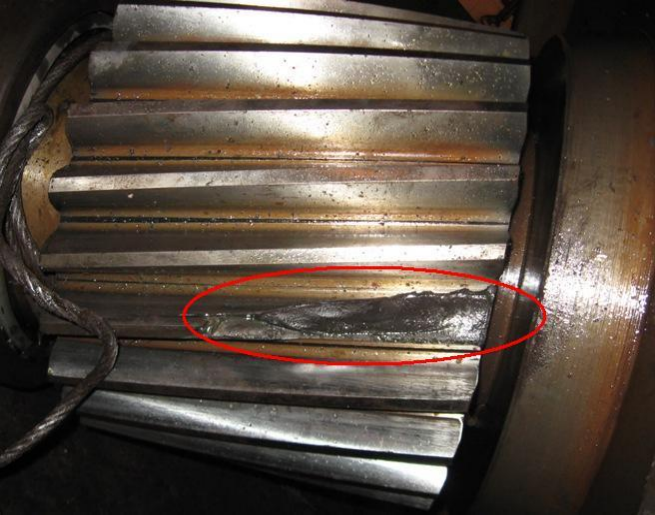
The real case of broken cog fault of Z5 (25-tooth) helical gear.

At 14:00 on Nov. 30th, 2008, through Fourier Transform and wavelet lifting analysis of the original vibration signals, both of these two methods show that the gear meshing frequency of Z5/Z6 in Shaft III in the sixth rack of rolling mill in some factory was 45.9 Hz. Figures 11(a,b) shows the original signal and the spectrum after Fourier Transform. Figures 11(c,d) shows the spectrum after autocorrelation analysis about the approximation signals which were obtained after wavelet transform reconstruction and decomposition to the data at level three. It can be seen that the SNR of the wavelet transform is higher in the spectrum analysis. The device disintegrated four days later; the broken cog tooth is shown in Figure 12.

#### 5.4.2. Case 2: fault diagnosis for broken cog

**Figure 13.**
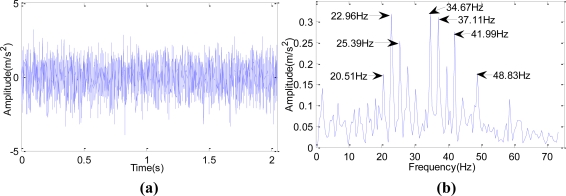

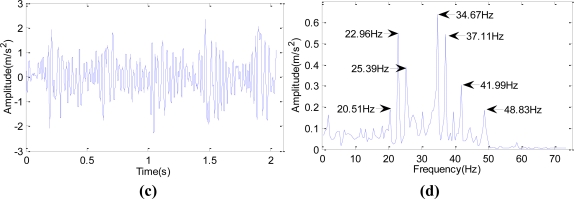
Comparison on spectrum of Fourier Transform and wavelet lifting analysis of the original signals. (a) time domain waveform of the original signal; (b) spectrum after Fourier transform of the original signal; (c) time domain waveform of approximation signal reconstruction after decomposition to wavelet lifting at level three; (d) spectrum analysis of wavelet lifting.

**Figure 14.**
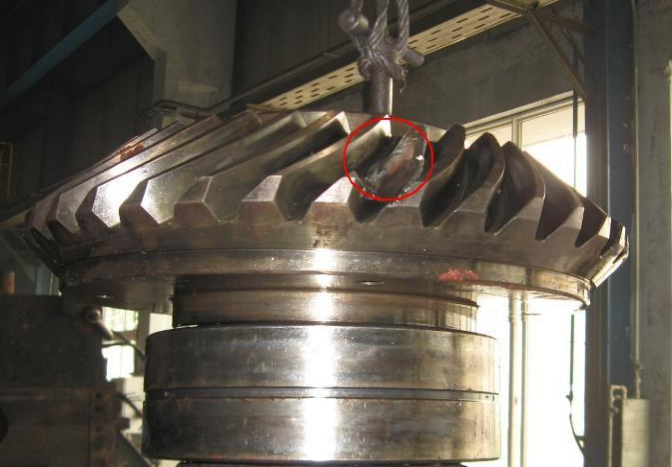
The real case of a broken cog fault of bevel gear Z2 (tooth 35).

At 4:00 on Jan. 25th, 2008, through Fourier Transform and wavelet lifting analysis of the original vibration signals at low frequency, it is found that the shaft-frequency of Shaft II in gear-box in the second rack of rolling mill was 2.44 Hz. Figures 13(a,b) shows the original signal and the spectrum after Fourier Transform. Figures 13(c,d) shows the spectrum after autocorrelation analysis of the approximation signals which were obtained after wavelet transform reconstruction and decomposition to the data at level three. It can be seen that the wavelet transform had more apparent features in spectrum and of higher SNR. The device was opened and checked 18 days later. The broken cog tooth is shown in Figure 14.

Some conclusions can be obtained through comparison of the above two cases, and are summarized in Table 3. It can be seen that the SNR of wavelet transform is higher, and the features extracted by wavelet transform is more apparent.

## 6. Conclusions

By using wavelet lifting, together with support vector machines and rule-based reasoning fault diagnosis methods, a real fault example of a broken cog in gearbox was analyzed and the following conclusions were drawn:

SVM is suitable for pattern recognition of problems with small sample sizes. In this study, two-class pattern recognition of actual gearbox faults was accomplished for diagnosis using SVM as the classifier. Based on the second generation wavelet packet feature extraction technology, by taking advantage of the fact that resonance occurs in the high frequency bands in the early stages of a fault, interference from noise signals from other frequency bands is effectively avoided through the decomposition and reconstruction of signals at high frequency bands; thus, fault feature extraction was achieved. According to the features of gearbox faults, a fuzzy production approach was applied to reveal fault rules, and rule-based reasoning was achieved through the fuzzy matrix. As demonstrated with actual data, this approach effectively overcomes the difficulty that some rules are difficult to present precisely.

Integrating different diagnosis technologies has become popular in intelligent diagnosis research. Taking advantage of each method in diagnosis inference such that the methods complement each other and create a hybrid diagnosis system is the goal for designing intelligent diagnosis technology.

## Figures and Tables

**Table 1.** The rules of knowledge.

**Fault cause**	Shaft imbalance	Shaft misalignment	Shaft bending	Foundation deformation	Gear Tooth profile error	Gear wear	Gear tooth breakage	Damage of bearing
***f_r_***	0.8	0.4	0.7	0.4	0.2	0	0.2	0.4
***2f_r_***	0	0.3	0	0.2	0.1	0	0.1	0.1
***3f_r_***	0	0.2	0	0.2	0.1	0	0	0.1
***6f_r_***	0	0	0	0	0	0	0	0.1
***9f_r_***	0	0	0	0	0	0	0	0.1
***f_m_***	0	0	0	0	0.2	0.4	0.2	0
***2f_m_***	0	0	0	0	0.1	0.2	0.1	0
**3*f_m_***	0	0	0	0	0.1	0.2	0.1	0
***x_q_ >3***	0	0	0	0	0.1	0.1	0.2	0.2
**Radial vibration direction**	0.2	0	0.3	0	0.1	0.1	0	0
**Axial vibration direction**	0	0.1	0	0.2	0	0	0	0
***f_m_* divided by peak value < 0.4**	0	0	0	0	0	0	0	0

**Table 2.** Ratios of different frequency components to the feature frequency.

**Type**	**Calculated frequency(Hz)**	**Feature frequency(Hz)**	**Ratio**
*f_m_*	1,140.00	1,139.277	0.999
*f_r_*	37.00	36.751	0.993

**Table 3.** Improving SNR and extracting features ability compared between Fourier transform (FT) and wavelet transform (WT).

**Case**	**Fault type**	**Position**	**Effect of noise reduction**		**Ability of picking-up features**	
			**FT**	**WT**	**FT**	**WT**
Case 1	Broken cog fault of helical cylindrical gear	Gear-box of blooming mill	Non-reduction	Mean-square difference *σ*/SNR *S* 1.02/0.33	General	Good
Case 2	Broken cog fault of bevel gear	Gear-box of blooming mill	Non-reduction	Mean-square difference *σ*/SNR *S* 4.20/0.13	General	Good
